# Nanoparticle separation with a miniaturized asymmetrical flow field-flow fractionation cartridge

**DOI:** 10.3389/fchem.2015.00045

**Published:** 2015-07-22

**Authors:** David Müller, Stefano Cattaneo, Florian Meier, Roland Welz, Andrew J. de Mello

**Affiliations:** ^1^Centre Suisse d'Electronique et de MicrotechniqueLandquart, Switzerland; ^2^Department for Chemistry and Applied Biosciences, Institute for Chemical and Bioengineering, ETH ZürichZürich, Switzerland; ^3^Postnova Analytics GmbHLandsberg am Lech, Germany

**Keywords:** nanoparticle separation, field flow fractionation, mAF4, miniaturization, sensitivity

## Abstract

Asymmetrical Flow Field-Flow Fractionation (AF4) is a separation technique applicable to particles over a wide size range. Despite the many advantages of AF4, its adoption in routine particle analysis is somewhat limited by the large footprint of currently available separation cartridges, extended analysis times and significant solvent consumption. To address these issues, we describe the fabrication and characterization of miniaturized AF4 cartridges. Key features of the down-scaled platform include simplified cartridge and reagent handling, reduced analysis costs and higher throughput capacities. The separation performance of the miniaturized cartridge is assessed using certified gold and silver nanoparticle standards. Analysis of gold nanoparticle populations indicates shorter analysis times and increased sensitivity compared to conventional AF4 separation schemes. Moreover, nanoparticulate titanium dioxide populations exhibiting broad size distributions are analyzed in a rapid and efficient manner. Finally, the repeatability and reproducibility of the miniaturized platform are investigated with respect to analysis time and separation efficiency.

## Introduction

**Figure 1:**
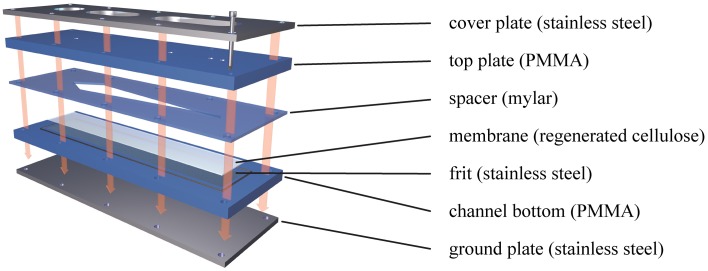
**Basic setup of an AF4 separation cartridge**. The shape of the separation channel is defined by the Mylar foil cutout. When all parts are screwed together, the pressure exerted on the edges of the foil pushes them into the membrane and seals the channel. The top plate consists of a solid PMMA sheet with fluidic connections for sample inlet and outlet. The channel bottom contains the frit, supporting the membrane and relaying the fluid pushed through the membrane toward the outlet crossflow. The steel plates on both sides of the assembly are used to compress the chip and withstand pressures in excess of 10 bar.

Having found applications in a large range of consumer products, nanoparticles have become an essential part of everyday life (Becker et al., 2009). A comprehensive characterization of engineered nanoparticles (ENPs) in terms of particle size, shape and chemistry is needed to ensure a consistent product quality. A common technique used to characterize ENPs is Asymmetrical Field Flow-Field Fractionation (AF4). For example, AF4 has been successfully applied to the analysis of gold (Schmidt et al., 2011), silver (Hagendorfer et al., 2012) and a range of non-metallic nanoparticles (Heroult et al., 2014). AF4 is applicable to the separation of particles over a wide size range (between 1 nm and 10 μm). Briefly, a suspension of sample particles is pumped into a long (~30 cm), narrow (~1 cm) and shallow (a few hundred microns) ribbon-like channel. In the classical, symmetrical Flow Field-Flow Fractionation (F4) format, a crossflow penetrating both upper and lower channel walls is applied perpendicularly to the general direction of flow (Giddings et al., 1976). In the asymmetrical version (AF4), the channel is enclosed by a solid top plate with fluid connectors and a bottom plate carrying a semipermeable membrane placed above a frit (Figure 1).

**Figure 2:**
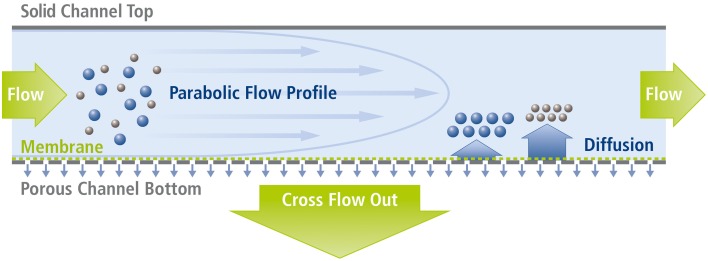
**Separation principle in Asymmetrical Field Flow-Field Fractionation (AF4)**. Smaller particles with higher diffusion coefficients stabilize (on average) further away from the membrane toward which they are drawn by the permanent crossflow. Thereby, small particles are subjected to faster streamlines than larger ones and accordingly exit the channel more quickly.

This design is technically simpler than the symmetrical approach, as it eliminates difficulties associated with heterogeneity and variable permeability of the upper frit, whilst still providing the flow gradients necessary for particle separation (Wahlund and Giddings, 1987). In the initial focusing step, a second pump (the so-called focus pump) is used to introduce a second inflow of eluent through the outlet end of the channel, creating two opposing flow streams meeting at a focusing point. Thereby, the injected particles within the fluid stabilize at different average heights above the membrane, where the down-force of the perpendicular crossflow is counterbalanced by the size-related diffusion properties of the particles (Giddings et al., 1978). Particles of different size are then subjected to different streamlines within the parabolic flow profile and separated according to size as they move through (and elute) the channel (Figure 2).

Despite the many advantages of AF4, its adoption for routine use has been limited by the large footprint of the separation cartridge, extended analysis times and excessive reagent consumption. The miniaturization of AF4 separation cartridges has therefore been the subject of much recent interest, due to the potential benefits of decreased sample requirements, reduced consumption of the mobile phase, faster separation times and a facile integration with both upstream and downstream analytical processing. The miniaturization of an AF4 module was first reported in 2004 (Kang and Moon, 2004), although this work primarily focused on a specific embodiment of AF4; the so-called frit inlet AF4 (Stevenson et al., 1999). In this study, the length and width of an AF4 channel was reduced by a factor of ~3 compared to macroscale systems, shrinking the total channel area from almost 40 cm^2^ to less than 5 cm^2^. In the following years, various miniaturized AF4 cartridges without inlet frits were applied to studies of lipoprotein aggregations (Yohannes et al., 2006) and the separation of biological vesicles (Oh et al., 2007). In 2011, Kim and Moon replaced the ceramic frits and clamping plates with thicknesses of several centimeters by 1.5 mm thick stainless steel plate components, obtaining a miniaturized cartridge resembling for the first time a planar fluidic device representative of Lab-on-a-Chip (LOC) or Micro Total Analysis Systems (μTAS) (Kim and Moon, 2011). All of the above investigations reported good separation efficiencies, rapid elution times and lower sample/eluent consumptions coupled with lower output flowrates (which is especially important for subsequent analysis by Mass Spectrometry), but none provided information on the experimental robustness and repeatability of the miniaturized cartridges; critical parameters in the industrial application of the core technology. Accordingly, we present herein a widely applicable miniaturized AF4 cartridge, which is comprehensively characterized in terms of separation efficiency and reproducibility of peak position and shape, using certified gold and silver nanoparticle standards, as well as commercially relevant nanoparticulate titanium dioxide samples with broad size distributions.

## Materials and methods

### Chemicals and reagents

Gold nanoparticle standards were purchased from the National Institute of Standards and Technology (NIST RM® 8011, 8012, and 8013). Silver nanoparticle standards (EM.SC20 and EM.SC60) were obtained from BBI (BBI Solutions, UK). Nanoparticulate TiO_2_ (AERODISP® w740x, Evonik Industries, Germany) was obtained as a highly concentrated suspension (40%, w/v). Prior to analysis, all standard suspensions were diluted to suitable concentrations in the respective eluent (details regarding concentrations and mixing ratios are provided in the Supplementary Data Sheet 1) and subsequently placed in an ultrasonic bath (Sonorex Digital 10 P, Bandelin, Berlin, Germany) at maximum power for 30 min. For the Au nanoparticle analysis, filtrated ultrapure water (MilliQ, Billerica, USA) was used. The eluent for TiO_2_ analysis was also prepared using filtrated ultrapure water, to which 0.05% (v/v) filtered NovaChem® (Postnova Analytics GmbH, Germany) was added. Eluent for the analysis of the Ag nanoparticles was again filtrated ultrapure water with 0.05% (v/v) filtered NovaChem®, which was adjusted to pH 9.2 with 0.1 M NaOH.

### Instrumentation

All AF4 measurements were performed using a commercially available AF2000 MF Flow FFF system from Postnova Analytics GmbH (PN), Germany, including an autosampler (PN5300), UV (PN3211) and Multi-Angle Light Scattering MALS (PN3621) detectors. UV detection was performed at 254 nm (TiO_2_), 470 nm (Ag) and 530 nm (Au). The MALS detector yielded information regarding the particle size distribution of the TiO_2_ nanoparticle population. Evaluation of the newly developed miniaturized AF4 channel cartridge (mAF4 channel) was performed by direct comparison to an instrument equipped with Postnova's standard analytical AF4 cartridge (S-AF4-CHA-611) incorporating a 10 kDa regenerated cellulose membrane (Z-AF4_MM-612-10KD). Data acquisition was performed using the AF2000 Control Unit software (Postnova Analytics GmbH, Germany) and further evaluations (such as the Gaussian curve fitting and statistical analyses shown in Table 1) were done using OriginPro 2015 (OriginLab Corporation, USA). Operational parameters with respect to injection volumes, volumetric flow rates and analysis times are provided in the Supplementary Data Sheet 1.

**Table 1:** **Statistical analysis of the experimental reproducibility of the miniaturized cartridge**.

	**20 nm Ag-NP**		**60 nm Ag-NP**		**AERODISP TiO**_**2**_**-NP**	
	**Average (min)**	**St. Dev**.	**Average (min)**	**St. Dev**.	**Average (min)**	**St. Dev**.
Peak maximum	7.31	0.43	16.15	0.22	8.58	0.11
Peak center, fitted curve	7.13	0.18	16.11	0.14	8.72	0.10
Fitted curve FWHM	1.21	0.10	2.50	0.10	2.6	0.04

### Miniaturized AF4 cartridge

**Figure 3:**
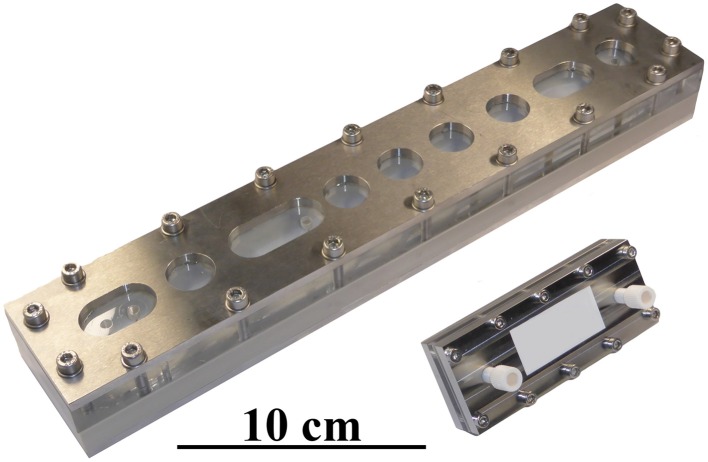
**Image of a miniaturized Asymmetrical Field Flow-Field Fractionation (mAF4) cartridge and a commercially available analytical AF4 channel**. The smaller size of the mAF4 cartridge makes it more economical and results in shorter analysis times. It is also easier to handle, thereby speeding up general maintenance tasks, such as exchanging the channel membrane.

To ensure a wide applicability of the developed cartridge, the miniaturized separation module was conceptually similar in design to commercially available macroscopic analytical channels, such as the Analytical AF4 Cartridge from Postnova Analytics GmbH, Germany. The footprint of the complete cartridge was 38×95 mm^2^, with the stainless steel frit being 20×80 mm^2^. The shape of the channel in the Mylar foil cut-out was similar to the standard channel (trapezoidal), but with a reduced tip-to-tip length of 70 mm, an initial width of 10 mm and an end width of 5 mm. The completed assembly is shown in Figure 3, alongside a standard AF4 analytical channel, and contains two fluidic connectors on the cover and a crossflow output on the ground plate. One of the fluidic connections on the cover plate was used exclusively for the sample/eluent input, with the other being coupled to a four-way PEEK cross connector (P-723, ERCATECH AG, Switzerland), which was further connected to the focus pump, a purge valve (to flush and clean the system) and the UV and MALS detectors.

## Results and discussion

### Separation performance

**Figure 4:**
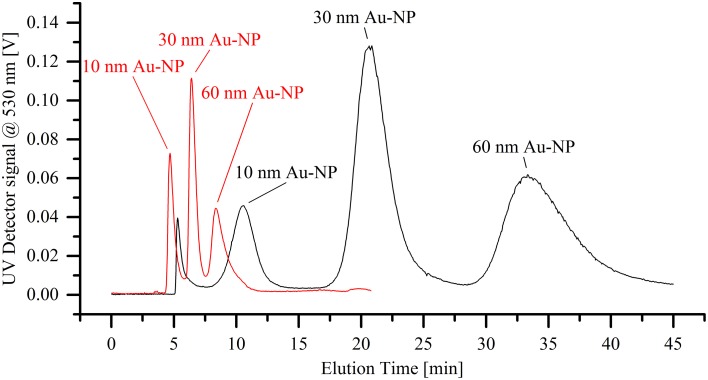
**Separation of a dispersion of differently sized gold nanoparticle standards (10, 30, and 60 nm in diameter)**. The black line shows the elugram generated when using the standard analytical cartridge, whereas the red line demonstrates the significantly faster separation through the miniaturized cartridge.

The separation efficiency of the miniaturized cartridge was initially assessed by analyzing a mixture of gold nanoparticle standards with diameters of 10, 30, and 60 nm. Figure 4 shows the separation of those three particle species into distinct peaks. It is observed that miniaturization of the separation cartridge reduces the measurement time by a factor of 4, whilst still allowing a clear separation of the three size populations. In order to prevent sample overloading, the injected sample concentration was reduced by 75 % compared to that used in the analytical channel. Interestingly, maximal signal intensities associated with the miniaturized cartridge in Figure 4 are comparable or even superior to those obtained using the conventional system. This can be explained by the narrower peak widths, with separated fractions passing through the detector within a shorter time period (resulting in a higher concentration of particles within the detection volume per unit time) and thus providing better signal-to-noise (S/N) ratios. For this experiment, the S/N ratio increases by a factor of 5.3 for the 10 nm AuNP peak, and 2.6 and 2.3 for the 30 nm and 60 nm AuNP peaks, respectively. However, it should be noted that these values are highly method dependent and in this case the separation of gold nanoparticles using the micro cartridge does not display a baseline separation, as observed for the analytical cartridge. A method change toward a baseline separation on the miniaturized cartridge would likely increase separation times and thus lead to decreased signal-to-noise ratios. It is also significant that essentially no void peak is observed when using the miniaturized cartridge. This is likely to be a consequence of the higher flux of eluent through the membrane. Although the overall active membrane area was reduced by a factor of 7.5, the crossflow was only slightly reduced (in this case from a 1.0 mL/min to 0.7 mL/min), resulting in a significantly higher flux through the membrane.

**Figure 5:**
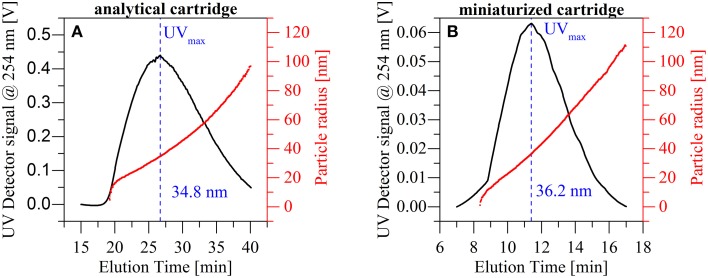
**Comparison of the particle size distribution obtained with the standard analytical cartridge (A) and the miniaturized AF4 cartridge (B) for a titanium dioxide dispersion**. The results show similar particle distributions and a similar particle size at UV_max_.

In additional experiments, we evaluated the reproducibility with respect to peak shape and position in the mAF4 cartridge when compared to the standard AF4 cartridge in a more complex sample. This was achieved through the separation of nanoparticulate TiO_2_ and simultaneous measurement of the mean particle radius via Multi Angular Light Scattering (MALS) (Figure 5). As a reference point for particle size, the highest concentration identified by the UV absorption detector (UV_max_) was selected. The overall peak shape obtained using both cartridges was highly comparable as was the size distribution across the full TiO_2_-peak. MALS-data from the standard cartridge indicate a mean particle radius of 34.8 nm at the maximum UV signal, compared to the 36.2 nm obtained using the miniaturized cartridge. This percentage deviation is well below 5% and therefore fully within the expected values.

### Channel reproducibility and robustness

**Figure 6:**
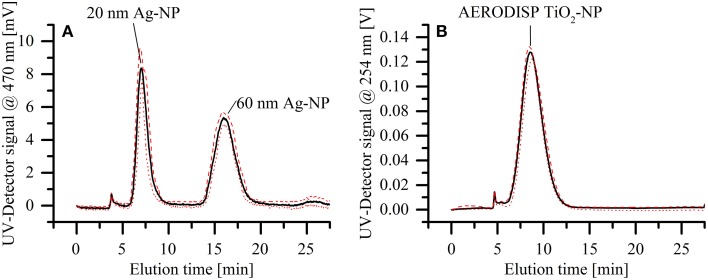
**Assessment of reproducibility for the mAF4 channel**. The solid black lines show the average of all measurements, whereas the red dotted and red dashed lines show the minimal and maximal values respectively. **(A)** Separation of a dispersion of two different silver nanoparticle standards (20 and 60 nm in diameter). The elution was repeated on different membranes and during different days within a 2-week interval. **(B)** Elution of TiO_2_ nanoparticle dispersion, repeated eight times in quick succession.

The reproducibility of separations using the miniaturized cartridge was assessed using an elution series for a mixture of two silver nanoparticle standards (20 and 60 nm in diameter). The dispersion was separated six times over the course of 2 weeks, using three different membranes of the same batch (10 kDa, regenerated cellulose). By exchanging the membrane in between measurements, we demonstrated that such regular maintenance operation does not have a significant influence on the separation quality. Figure 6A shows the average of all measurements (solid black line). In addition, the minimal (red dotted line) and maximal (red dashed line) values for each time segment are displayed, showing only minor variability over the course of the entire separation. The reproducibility of the setup was further tested by eluting the AERODISP TiO_2_ nanoparticles eight times in quick succession. The slight deviations evident from Figure 6B are expected in AF4 separations, especially when using a fresh membrane. A statistical analysis of these three peaks is provided in Table 1. To assess the robustness of our system, reproducibility measurements were performed on identical systems, but in different laboratories; specifically, separation of silver particles was performed at the CSEM laboratories in Landquart, Switzerland, while the titanium dioxide measurements were performed at the European Application Center of Postnova Analytics GmbH in Germany.

## Concluding remarks

Miniaturized separation cartridges for AF4 can offer several advantages over conventional and commercially available cartridges, including significantly reduced analysis times and reduced solvent consumption. Due to reduced sample dilution (as a result of minimal band dispersion), a high sensitivity and improved signal to noise ratios are attainable. However, to have a significant impact in practical applications such advantages must be accompanied by a high degree of analytical repeatability and reproducibility.

In the current study, we developed and characterized a miniaturized AF4 cartridge suitable for wide application, as a first step toward a fully validated and field-independent analytical platform. The performance of the miniaturized AF4 system was characterized thoroughly using Au and Ag nanoparticle standards, as well as TiO_2_ particle mixtures possessing a wide size distribution. The newly developed cartridge showed a separation performance comparable to or even better than standard, macroscopic AF4 cartridges, while significantly reducing the analysis time. In case of the Au nanoparticles, a sensitivity increase of 2–5 times (depending on particle size) was achieved. In conclusion, mAF4 has the potential to be a time- and cost-saving alternative to established AF4 separation cartridges. It should be noted that the reduced sample capacity of the mAF4-cartridge does not impact its utility in analytical separations, but does set limits for its use in preparative applications. Further hardware adaptations, such as a third fluidic connector in the cover plate, will make such a miniaturized approach even more versatile. This modification would allow splitting the outlet stream (Giddings et al., 1983), thus enabling a further improvement of the sensitivity of the system, as it is already known from standard cartridges (Prestel et al., 2006).

## Conflict of interest statement

The authors declare that the research was conducted in the absence of any commercial or financial relationships that could be construed as a potential conflict of interest.
