# Supplementary material for: Nanoparticle separation with a miniaturized asymmetrical flow field-flow fractionation cartridge
Source: Front Chem. 2015 Jul 22;3:45. doi: 10.3389/fchem.2015.00045 (PMC4510429; doi:10.3389/fchem.2015.00045)
Supplement: Supplementary file 1 [file DataSheet1.DOCX]

***Supplementary Material***

**Nanoparticle separation with a miniaturized Asymmetrical Flow Field-Flow Fractionation (mAF4) cartridge**

**David Müller^1,3^*, Stefano Cattaneo^1^, Florian Meier^2^, Roland Welz^2^, Andrew de Mello^3^**

^1^Centre Suisse d’Electronique et de Microtechnique (CSEM), Landquart, Switzerland

^2^Postnova Analytics GmbH, Landsberg am Lech, Germany

^3^Institute for Chemical and Bioengineering, Department for Chemistry and Applied Biosciences, ETH Zürich, Switzerland

*** Correspondence:** David Müller, Institute for Chemical and Bioengineering, Department for Chemistry and Applied Biosciences, ETH Zürich, Vladimir-Prelog-Weg 1, 8093 Zürich, Switzerland.

david.mueller@chem.ethz.ch

1. **Specific separation protocols**

Supplementary Table 1: Sample information and the AF4 method parameters of the standard analytical cartridge measurement shown in Figure 4 (black line).

| Sample: | Gold nanoparticle standards: 10, 30 and 60 nm in diameter from the National Institute of Standards and Technology (NIST RM® 8011, 8012 and 8013). | | |
| --- | --- | --- | --- |
| Concentrations: | 10 nm Au-NP, NIST RM® 8011: 51.56 ± 0.23 µg mL^-1^ 30 nm Au-NP, NIST RM® 8012: 48.17 ± 0.33 µg mL^-1^ 60 nm Au-NP, NIST RM® 8013: 51.86 ± 0.64 µg mL^-1^ | | |
| Mixing and dilution: | Mixing ratio was 1:1:1, sample was not diluted. | | |
| Injection volume: | 20 µl | | |
| Eluent: | Ultrapure water (MilliQ, Billerica, USA) | | |
|  |  |  | |
| Injection / Focusing |  |  |  |
| Detector flow rate (mL min^-1^) | 0.30 | Cross flow rate (mL min^-1^) | 1.00 |
| Injection flow rate (mL min^-1^) | 0.20 | Injection time (min) | 4.0 |
| Focus flow rate (mL min^-1^) | 1.10 | Transition time (min) | 0.2 |
|  |  |  |  |
| 1^st^ elution step |  |  |  |
| Elution time (min) | 40.0 | Initial cross flow (mL min^-1^) | 1.0 |
| Elution type | constant |  |  |
|  |  |  |  |

Supplementary Table 2: Sample information and the mAF4 method parameters of the miniaturized cartridge measurement shown in Figure 4 (red line).

| Sample: | Gold nanoparticle standards: 10, 30 and 60 nm in diameter from the National Institute of Standards and Technology (NIST RM® 8011, 8012 and 8013). | | |
| --- | --- | --- | --- |
| Concentrations: | 10 nm Au-NP, NIST RM® 8011: 51.56 ± 0.23 µg mL^-1^ 30 nm Au-NP, NIST RM® 8012: 48.17 ± 0.33 µg mL^-1^ 60 nm Au-NP, NIST RM® 8013: 51.86 ± 0.64 µg mL^-1^ | | |
| Mixing and dilution: | Mixing ratio was 1:1:1, diluted by a factor 4 with ultrapure water (MilliQ, Billerica, USA). | | |
| Injection volume: | 20 µl | | |
| Eluent: | Ultrapure water (MilliQ, Billerica, USA) | | |
|  |  |  |  |
| Injection / Focusing |  |  |  |
| Detector flow rate (mL min^-1^) | 0.50 | Cross flow rate (mL min^-1^) | 0.70 |
| Injection flow rate (mL min^-1^) | 0.20 | Injection time (min) | 2.5 |
| Focus flow rate (mL min^-1^) | 1.00 | Transition time (min) | 1.0 |
|  |  |  |  |
| 1^st^ elution step |  |  |  |
| Elution time (min) | 15.0 | Initial cross flow (mL min^-1^) | 0.70 |
| Elution type | power | Exponent | 0.4 |
|  |  |  |  |
| 2^nd^ elution step |  |  |  |
| Elution time (min) | 2.5 | Initial cross flow (mL min^-1^) | 0.00 |
| Elution type | constant |  |  |

Supplementary Table 3: Sample information and the AF4 method parameters of the standard analytical cartridge measurement shown in Figure 5A.

| Sample: | Titanium dioxide nanoparticle (AERODISP® w740x, Evonik Industries, Germany) | | |
| --- | --- | --- | --- |
| Concentration: | TiO_2_-NP,: 400.0 ± 10.0 mg mL^-1^ | | |
| Mixing and dilution: | Diluted by a factor 2000 with ultrapure water (MilliQ, Billerica, USA). | | |
| Injection volume: | 20 µl | | |
| Eluent: | Ultrapure water (MilliQ, Billerica, USA), to which 0.05% (v/v) filtered NovaChem® (Postnova Analytics GmbH, Germany) was added. | | |
|  |  |  |  |
| Injection / Focusing |  |  |  |
| Detector flow rate (mL min^-1^) | 0.50 | Cross flow rate (mL min^-1^) | 1.00 |
| Injection flow rate (mL min^-1^) | 0.20 | Injection time (min) | 7.0 |
| Focus flow rate (mL min^-1^) | 1.30 | Transition time (min) | 0.5 |
|  |  |  |  |
| 1^st^ elution step |  |  |  |
| Elution time (min) | 5.0 | Initial cross flow (mL min^-1^) | 1.00 |
| Elution type | constant |  |  |
|  |  |  |  |
| 2^nd^ elution step |  |  |  |
| Elution time (min) | 40.0 | Initial cross flow (mL min^-1^) | 1.00 |
| Elution type | power | Exponent | 0.2 |

Supplementary Table 4: Sample information and the mAF4 method parameters of the miniaturized cartridge measurement shown in Figure 5B.

| Sample: | Titanium dioxide nanoparticle (AERODISP® w740x, Evonik Industries, Germany) | | |
| --- | --- | --- | --- |
| Concentration: | TiO_2_-NP,: 400.0 ± 10.0 mg mL^-1^ | | |
| Mixing and dilution: | Diluted by a factor 2000 with ultrapure water (MilliQ, Billerica, USA). | | |
| Injection volume: | 5 µl | | |
| Eluent: | Ultrapure water (MilliQ, Billerica, USA), to which 0.05% (v/v) filtered NovaChem® (Postnova Analytics GmbH, Germany) was added. | | |
|  |  |  |  |
| Injection / Focusing |  |  |  |
| Detector flow rate (mL min^-1^) | 0.40 | Cross flow rate (mL min^-1^) | 0.25 |
| Injection flow rate (mL min^-1^) | 0.15 | Injection time (min) | 4.0 |
| Focus flow rate (mL min^-1^) | 0.50 | Transition time (min) | 0.5 |
|  |  |  |  |
| 1^st^ elution step |  |  |  |
| Elution time (min) | 4.0 | Initial cross flow (mL min^-1^) | 0.25 |
| Elution type | constant |  |  |
|  |  |  |  |
| 2^nd^ elution step |  |  |  |
| Elution time (min) | 20.0 | Initial cross flow (mL min^-1^) | 0.25 |
| Elution type | power | Exponent | 0.2 |

Supplementary Table 5: Sample information and the mAF4 method parameters of the miniaturized cartridge measurement shown in Figure 6A.

| Sample: | Silver nanoparticle standards: 20 and 60 nm in diameter from BBI Solutions, UK. | | |
| --- | --- | --- | --- |
| Concentrations: | 20 nm Ag-NP, EM.SC20: 7.00E+10 particles mL^-1^ 60 nm Ag-NP, EM.SC60: 2.60E+09 particles mL^-1^ | | |
| Mixing and dilution: | Mixing ratio was 1:1, diluted by a factor 10 with ultrapure water (MilliQ, Billerica, USA). | | |
| Injection volume: | 20 µl | | |
| Eluent: | Ultrapure water (MilliQ, Billerica, USA), to which 0.05% (v/v) filtered NovaChem® (Postnova Analytics GmbH, Germany) was added and which was adjusted to pH 9.2 with 0.1 M NaOH. | | |
|  |  |  |  |
| Injection / Focusing |  |  |  |
| Detector flow rate (mL min^-1^) | 0.30 | Cross flow rate (mL min^-1^) | 0.70 |
| Injection flow rate (mL min^-1^) | 0.20 | Injection time (min) | 2.5 |
| Focus flow rate (mL min^-1^) | 0.80 | Transition time (min) | 1.0 |
|  |  |  |  |
| 1^st^ elution step |  |  |  |
| Elution time (min) | 20 | Initial cross flow (mL min^-1^) | 0.70 |
| Elution type | power | Exponent | 0.4 |
|  |  |  |  |
| 2^nd^ elution step |  |  |  |
| Elution time (min) | 25.0 | Initial cross flow (mL min^-1^) | 0.00 |
| Elution type | constant |  |  |

Supplementary Table 6: Sample information and the mAF4 method parameters of the miniaturized cartridge measurement shown in Figure 6B.

| Sample: | Titanium dioxide nanoparticle (AERODISP® w740x, Evonik Industries, Germany) | | |
| --- | --- | --- | --- |
| Concentration: | TiO_2_-NP,: 400.0 ± 10.0 mg mL^-1^ | | |
| Mixing and dilution: | Diluted by a factor 2000 with ultrapure water (MilliQ, Billerica, USA). | | |
| Injection volume: | 5 µl | | |
| Eluent: | Ultrapure water (MilliQ, Billerica, USA), to which 0.05% (v/v) filtered NovaChem® (Postnova Analytics GmbH, Germany) was added. | | |
|  |  |  |  |
| Injection / Focusing |  |  |  |
| Detector flow rate (mL min^-1^) | 0.35 | Cross flow rate (mL min^-1^) | 0.30 |
| Injection flow rate (mL min^-1^) | 0.15 | Injection time (min) | 4.0 |
| Focus flow rate (mL min^-1^) | 0.50 | Transition time (min) | 0.5 |
|  |  |  |  |
| 1^st^ elution step |  |  |  |
| Elution time (min) | 1.5 | Initial cross flow (mL min^-1^) | 0.30 |
| Elution type | constant |  |  |
|  |  |  |  |
| 2^nd^ elution step |  |  |  |
| Elution time (min) | 20.0 | Initial cross flow (mL min^-1^) | 0.30 |
| Elution type | power | Exponent | 0.2 |
|  |  |  |  |
| 3^rd^ elution step |  |  |  |
| Elution time (min) | 10.0 | Initial cross flow (mL min^-1^) | 0.00 |
| Elution type | constant |  |  |
